# Glypican-3-Specific CAR NK Cells Co-Secreting IL-15 and IFN-α Have Increased Anti-Tumor Function Versus Hepatocellular Carcinoma In Vitro

**DOI:** 10.3390/ijms262411892

**Published:** 2025-12-10

**Authors:** Rosalia Busà, Gioacchin Iannolo, Bruno Douradinha, Duilio Pagano, Anna Gallina, Giancarlo Cappello, Antonio La Rocca, Salvatore Gruttadauria, Pier Giulio Conaldi, Ester Badami

**Affiliations:** 1 IRCCS ISMETT, 90127 Palermo, Italy; 2 Department of Medicine and Surgery, Kore University of Enna, 94100 Enna, Italy; 3 Fondazione Ri.MED, 90133 Palermo, Italy; 4 Department of Surgery and Medical and Surgical Specialties, University of Catania, 95124 Catania, Italy

**Keywords:** adoptive cell therapy, cancer immunotherapy, chimeric antigen receptor, genetic modification, natural killer cells, hepatocellular carcinoma, Glypican-3

## Abstract

Chimeric antigen receptor (CAR)-modified natural killer (NK) cells represent a promising immunotherapeutic approach for the treatment of oncological malignancies such as hepatocellular carcinoma (HCC). In this work, we have engineered primary human NK cells, re-directing them so they can specifically recognize Glypican-3 (GPC3), an immunotherapeutic target for HCC. In previous studies, we have demonstrated that IFN-α significantly enhances NK cells’ anti-tumor and anti-viral cytotoxicity. Fourth-generation self-inactivating lentiviral vectors were used to deliver a transgenic expression of IFN-α or its co-expression with IL-15 (which induces NK cells expansion, survival, and function), aiming to enhance CAR-GPC3 NK cells’ anti-tumor response against HCC. We optimized a protocol for efficient transduction of primary NK cells, demonstrating that CAR expression is maintained at high levels over time. Exposure of HCC ectopically expressing GPC3+ to CAR-GPC3-IL15 and CAR-GPC3-IL15-IFNα NK cells demonstrated significant in vitro cytotoxicity and cytokine production, dependent on GPC3 expression. To prevent undesired side effects of CAR-NK cell immunotherapy, co-delivery with a suicide gene is advised as a safety measure. Thus, a truncated epidermal growth factor receptor (tEGFR) was co-delivered with the anti-GPC3 CAR, which efficiently promoted the suicide of the CAR-NK used in this work. Our study demonstrates the efficacy of re-directed CAR-GPC3 primary NK cells, encouraging further preclinical and clinical translation studies and strengthening the potential of these cells as a novel treatment option for patients with HCC.

## 1. Introduction

Liver cancer is one of the leading global causes of cancer-related mortality, with hepatocellular carcinoma (HCC) being the most prevalent liver tumor type [1,2]. Notably, infection with hepatitis B virus and hepatitis C virus (HBV and HCV, respectively) constitutes the primary risk factors contributing to HCC development [3,4,5,6] followed closely by non-alcoholic fatty liver disease [7]. Unfortunately, only 40% of HCC cases receive early-stage diagnoses, and treatment outcomes are often less than satisfactory. While surgical intervention remains the preferred curative approach, only a minority of HCC tumors are amenable to resection. Furthermore, cancer recurrence is strikingly high, afflicting 50–70% of patients within five years post-surgery, for which liver transplantation represents a unique lifesaving treatment [8]. Emerging approaches aim to identify specific molecular targets to use as diagnostic biomarkers for early diagnosis or immunotherapeutic targets. One of the most studied is Glypican-3 (GPC3), a membrane-associated heparan sulfate proteoglycan implicated in cell growth, differentiation, and migration [9], highly expressed in at least 70% of HCC patients but not in normal adult tissues [10,11]. To date, there are many ongoing innovative GPC3-targeted therapeutic approaches, including anti-GPC3 immunotoxin, GPC3 vaccine, combined therapy with immune checkpoint inhibitors, and chimeric antigen receptor (CAR) T or NK-92 cells [12,13]. In particular, GPC3-targeted CAR-T cells were found to eliminate GPC3-positive HCC cells in mice [14] and patient-derived HCC xenograft models [15], further confirming the potential use of GPC3-specific CAR-T cells as a therapy for the treatment of HCC. More recently, different phase I clinical trial studies report encouraging initial safety data on the use of CAR-GPC3 T cell therapy for the treatment of advanced (NCT02395250, NCT05003895, [16]) and refractory GPC3-expressing HCC (NCT03146234). Despite the fact that CAR-T therapy has rapidly emerged as a promising clinical approach to treat cancer [15,17], among which is HCC [18], unfortunately, their use in clinical trials has been limited due to the occurrence of associated side effects including cytokine release syndrome (CRS) and neurotoxicity and especially because their use requires human leukocyte antigen (HLA) matching to prevent graft-versus-host disease (GvHD), increasing the complexity, the cost, and the time for autologous CAR-T manufacturing [19,20]. Unlike with T cell-based immunotherapy, natural killer (NK) cell function does not require antigen pre-sensitization or full HLA matching [21,22]. Moreover, they are specialized innate immune cells that, among other functions, exert potent cytotoxicity against cancer cells [23]. Also, CAR-NK cells are associated with improved efficacy and fewer side effects when compared to T cell-based therapies [24,25] and have been proven to be effective against cancer [26,27] and autoimmune disease [28].

Based on current knowledge, our study aimed to engineer primary CAR-NK cells to express the chimeric antigen receptor specific to the HCC-related tumor target Glypican-3. For that scope, we used two constructs that shared the same intracellular signaling region consisting of CD28, 4–1BB, CD3ζ, and a CD8α hinge domain. As a suicide gene, we chose a truncated form of the human epidermal growth factor receptor (tEGFR), which acts as a “safety switch” in vivo to remove CAR-NK cells with cetuximab, an FDA-approved antibody which is commercially available [29,30]. The two genetic plasmids used here can be classified as fourth-generation constructs since they were additionally engineered to secrete transgenic cytokines upon CAR signaling in the targeted tumor tissue. Both constructs lead to the secretion of IL-15, a cytokine that plays a critical role in the immune system as an essential mediator of NK cell peripheral homeostasis. IL-15 promotes the survival, proliferation, and function of NK cells by supporting their development, enhancing their cytotoxic activity, and preventing apoptosis. Engineered in CAR constructs, IL-15 has been proven to be effective in supporting and enhancing the anti-tumor cell function both in solid and blood cancers [31]. This cytokine also acts as a bridge between the innate and adaptive immune systems, contributing to the maintenance and activation of memory CD8+ T cells, while the signaling pathways are crucial for the regulation of immune responses [32]. Out of the two plasmids used in this work, one was modified to also secrete a second soluble cytokine, interferon-alpha (IFN-α). IFN-α is a type I interferon with potent immunomodulatory, antiviral, and anti-tumor properties. It acts by binding to specific receptors on the surface of immune and non-immune cells, triggering a signaling cascade that activates the transcription of numerous genes involved in innate and adaptive immunity, such as the STAT1/2 pathways. This cytokine is particularly effective in the enhancement of NK cell activity by increasing their cytotoxicity, promoting the expression of activating receptors, and improving their ability to produce pro-inflammatory cytokines. Furthermore, IFN-α plays a critical role in shaping the tumor microenvironment by upregulating MHC class I molecules on tumor cells, making them more visible to immune surveillance, and stimulating dendritic cells to prime adaptive immune responses. Previous studies have confirmed that IFN-α significantly boosts NK cell action against tumor cells and pathogens, both in vitro and in vivo, reinforcing its potential as a therapeutic agent in cancer and infectious diseases [33,34]. We characterized both the expression and function of the two CAR constructs, namely CAR-GPC3-IL15 and CAR-GPC3-IL15-IFNα, and provided evidence that supports the potential use of soluble cytokine IFN-α to enhance anti-GPC3-specific primary CAR-NK cell function in hepatocellular carcinoma.

## 2. Results

### 2.1. IFN-α-Activated NK Cells Have Enhanced Cytotoxicity Against Hepatocellular Carcinoma Cell Lines

**Figure 1:**
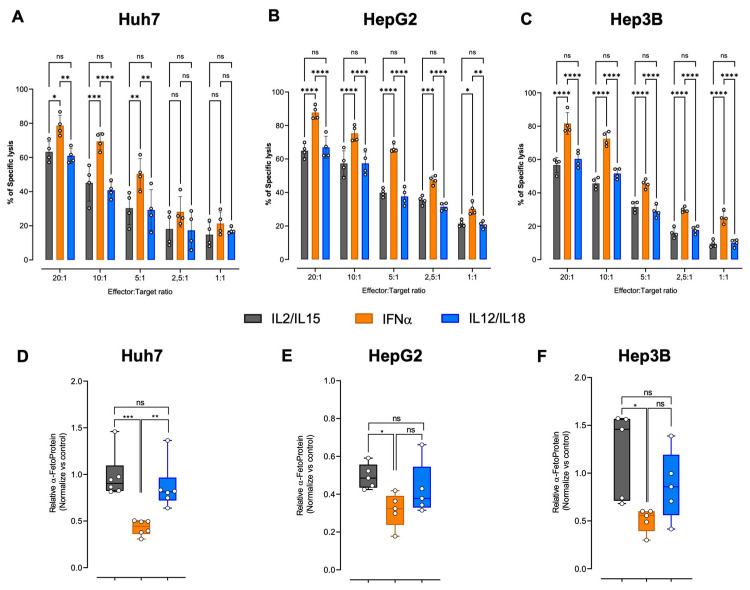
Functional characterization of NK cells activated with IL-2/IL-15, IFN-α, or IL-12/IL-18. (**A**–**C**) Percentage of lysed target Huh7 (**A**), HepG2 (**B**), or Hep3B target cells (**C**) following exposure to decreasing ratios (20:1, 10:1, 5:1, 2,5:1, 1:1) of IL2/IL15-NK (gray bars), IFNα-NK (orange bars), or IL12/IL18-NK cells (blue bars) as calculated with a classic four-hour chromium release assay. Each dot represents the average of three technical replicates from each donor (*n* = 4). Mean values ± SEM are shown. (**D**–**F**) Alpha-Fetoprotein was quantified on day seven in the culture medium of Huh7 (**D**), HepG2 (**E**), or Hep3B cells (**F**) co-cultured in transwell with IL2/IL15-NK, IFNα-NK, or IL12/IL18-NK cells. Each dot represents the average of three technical replicates from one donor (*n* = 5), and data are calculated as relative Alpha-Fetoprotein values normalized to the untreated control not co-cultured with NK cells. Mean values ± SEM are shown. Statistical analyses were performed using a two-way ANOVA with Tukey’s multiple comparisons test (**A**–**C**) and an ordinary one-way ANOVA with Tukey’s multiple comparisons test (**D**–**F**). * *p* < 0.05, ** *p* < 0.01, *** *p* < 0.001, **** *p* < 0.0001; ns, non-significant.

Several studies have shown that IFN-α treatment enhances cytokine secretion, polyfunctionality, degranulation, and the cytotoxic potential of NK cells [35,36,37,38]. In accordance with what has previously been demonstrated, we recently showed that IFNα-NK cells possess enhanced cytotoxic function against the human hepatoma cell line HepG2, especially in the early phases of the response, by inducing faster pore formation by target cells and consequent cell death [34]. Using this model, we observed an increased anti-viral response of IFNα-NK cells compared to IL2/IL15-NK cells, and this phenotype was also seen in a murine in vivo model [33,34]. To further characterize the role of cytokines in NK cell response to tumor cells, we pre-conditioned primary NK cells overnight with three different sets of cytokines, including IL-2/IL-15, which were important for maintenance and expansion of NK cells in vitro, IFN-α, a pro-inflammatory cytokine involved in both innate and adaptive immune responses [33,34,39], and IL-12/IL-18, reported to enhance anti-tumor function and induce “memory” properties [40]. Cytotoxicity of NK cells was tested against three common human hepatocellular carcinoma cell lines, including Huh7, HepG2, and Hep3B, and evaluated by a chromium release assay using different effector/target cells ratios (NK:HCC cell lines; 20:1; 10:1; 5:1; 2.5:1; 1:1). As shown in Figure 1A–C, we observed that activation with IFN-α induces the highest percentage increase in specific lysis against all cell lines studied, particularly on HepG2 and Hep3B in which we detected a high killing function even at lower ratios compared to both IL-2/IL-15- and IL-12/IL-18-activated NK cells. To assess the effect on tumor cell growth, we quantified, in the culture media, the levels of the liver tumor biomarker alpha-fetoprotein (AFP) [41]. As can be observed in Figure 1D–F, the levels of AFP were significantly reduced when the Huh7, HepG2, and Hep3B cell lines were co-cultured with IFNα-NK cells, whereas lower, albeit significant, reductions were obtained with IL2/IL15-NK and IL12/IL18-NK cells. Together, these data confirmed that IFN-α-enhanced anti-tumor NK cell function mediated both by cell–cell contact and by soluble factors.

### 2.2. Generation of Primary NK Cells Harboring Fourth-Generation GPC3-Specific CAR by Lentiviral Vector Transduction

**Figure 2:**
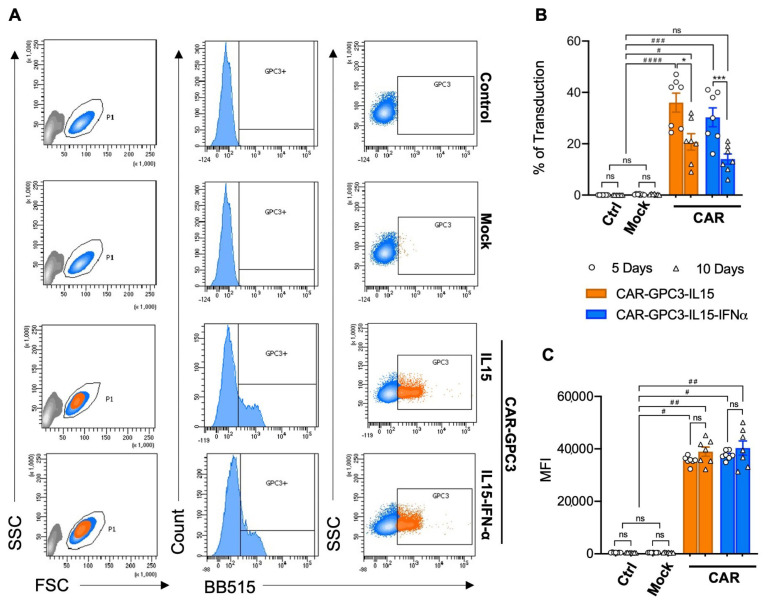
Generation of human primary NK cells harboring the fourth generation of GPC3-Specific CARs by lentiviral vector transduction. (**A**) Flow cytometric analysis of CAR expression on the surface of pure primary NK not transduced (control), transduced with lentiviral vector backbone (mock), transduced with CAR-GPC3-IL15, and GPC3-CAR-IL15-IFNα lentivirus with BB515-Labeled Human Glypican-3 protein. NK cells were gated on live cells according to morphology FSC/SSC. Data shown are representative of seven experiments with similar results. (**B**,**C**) Efficiency of transduction was traced on days 5 (dots) and 10 (triangles). Symbols (*n* = 7) represent the percentage of CAR-GPC3-IL15-NK (orange bars) and GPC3-CAR-IL15-IFNα-NK (blue bars) GPC3+ cells (**B**) and the mean fluorescence intensity (MFI, C). GFP (white bars) and mock (gray bars) were used as controls. (**B**,**C**) Statistical analyses were performed using a two-way ANOVA with the Kruskal–Wallis comparison test. * denotes significant intragroup differences (5 days vs. 10 Days), whereas # denotes significant differences between three major groups (Control, Mock and CARs). * *p* < 0.05, *** *p* < 0.001, # *p* < 0.05, ## *p* < 0.01, ### *p* < 0.001, #### *p* < 0.0001, ns non-significant.

In light of the results obtained by the exogenous stimulation of NK cells with IL-2/IL-15, IFN-α, or IL-12/IL-18 cytokines, we selected IFN-α and IL-15 to enhance NK cell cytotoxicity and survival [31], respectively. Hence, two fourth-generation CAR constructs were designed to engineer primary human NK cells specific to GPC3+ HCC. They both contained the full-length chimeric antigen receptor anti-GPC3 single-chain variable fragment (scFv) from antibody clone GC33, which was synthesized and subcloned into a self-inactivating LV6-EF1a-EGFRt-AT-free (cPPT) lentiviral vector backbone (Figure S1A,B), and inducible IL-15 cytokine. Importantly, only one of them also contained the gene for the secretion of IFN-α downstream of the CAR signaling region [38,42]. Briefly, the GPC3-CARs were composed of a signal peptide, a hinge region, a transmembrane domain, and the intracellular region, as extensively described in Figure S1C,D. Plasmids’ sizes were confirmed by diagnostic digestion, as shown in Figure S2. To optimize the cells’ transfection efficacy, we first transduced NK cells with a plasmid encoding a GFP reporter gene (GFP, Figure S3) and, subsequently, we transduced primary human NK cells using both GPC3-specific CAR constructs to generate CAR-GPC3-IL15 NK and CAR-GPC3-IL15-IFNα NK. Transduction efficiency and the correct folding of the CAR on NK cells’ surfaces was tested by flow cytometry using cognate protein Glypican-3 conjugated to a fluorochrome. Parental NK cells and transduction with empty vector (mock) were used as controls. Flow cytometry analysis in Figure 2A revealed that both GPC3-specific CARs were successfully integrated into the membrane of NK primary cells with comparable transduction rates for both lentiviral constructs. Cumulative flow cytometer analysis on seven different human NK cells revealed that, 5 days post-transduction, the mean transduction rate with CAR-GPC3-IL15 was slightly higher compared to CAR-GPC3-IL15-IFNα NK cells, with 36% ± 3.7 mean transduction rate versus 30.3% ± 3.7, respectively. Moreover, the transgene expression persists over time after transduction in both CAR-NK cells, although a decrease was observed at day 10 from 36% to 20.7% ± 3.2 for CAR-GPC3-IL15 and from 30.3% to 14% ± 2 for CAR-GPC3-IL15-IFNα NK cells (Figure 2B). Despite the reduction in the number of CAR+ NK cells, the mean fluorescence intensity (MFI) was maintained at a high value over time on days 5 and 10, being 35,532 ± 618 MFI and 37,576 ± 635 MFI on day 5 and 38,932 ± 1760 MFI and 40,329 ± 2696 MFI on day 10 in CAR-GPC3-IL15 and CAR-GPC3-IL15-IFNα NK cells, respectively (Figure 2C).

### 2.3. Lentiviral Transduction with Both GPC3-Specific CARs Enhanced the Cytotoxicity Effects of Primary Human NK

**Figure 3:**
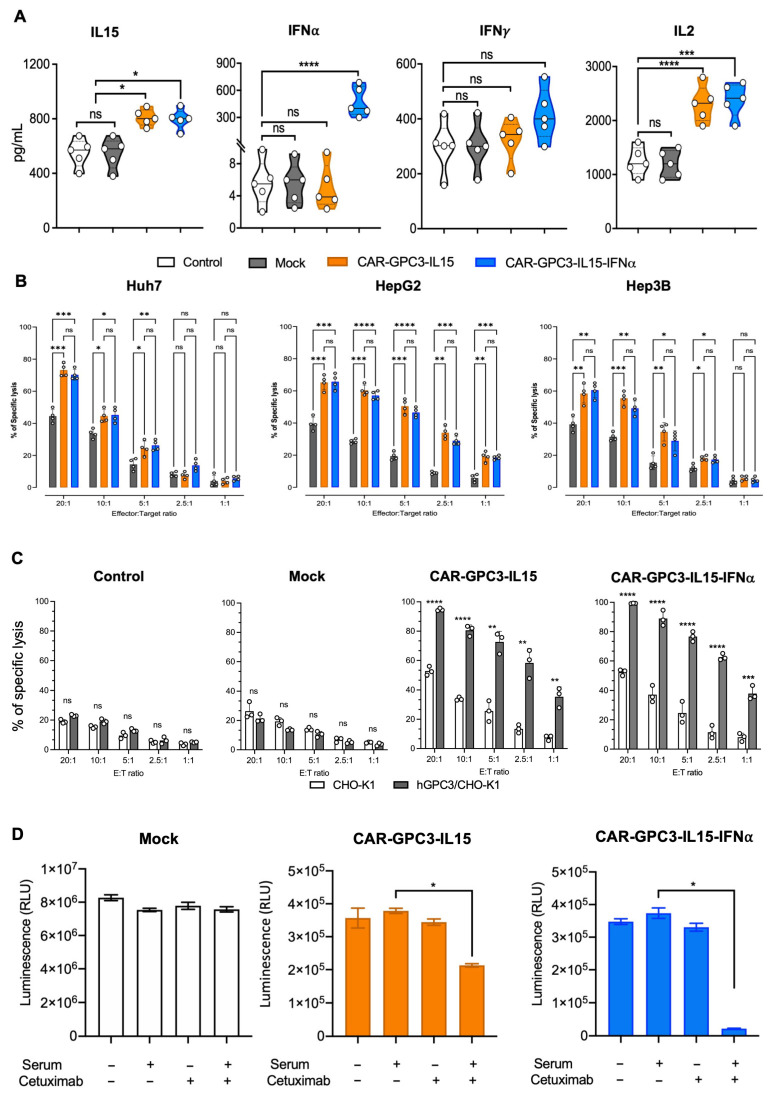
Characterization of CAR-GPC3-IL15-NK and GPC3-CAR-IL15-IFNα-NK cells. (**A**) IL-15, IFN-α, IFN-γ and IL-2 production by control, (white), mock (gray), CAR-GPC3-IL15 (orange), and GPC3-CAR-IL15-IFNα NK cells (blue) after 4 h of culture with Huh7 target cells. (**B**) Percentage of lysed target Huh7, HepG2, or Hep3B target cells following exposure to decreasing ratios (20:1, 10:1, 5:1, 2.5:1, 1:1) of mock control (gray bars), CAR-GPC3-IL15 (orange bars), and CAR-GPC3-IL15-IFNα NK cells (blue bars) as calculated with a four-hour chromium release assay. (**C**) Percentage of lysed target CHO-K1 (white bars), and hGPC3/CHO-K1 (gray bars) following exposure to decreasing ratios (20:1, 10:1, 5:1, 2,5:1, 1:1) of control, mock, CAR-GPC3-IL15, and CAR-GPC3-IL15-IFNα NK cells as calculated with a four-hour chromium release assay. Each dot represents the average of three technical replicates from one donor ((**A**), *n* = 5), ((**B**), *n* = 4), ((**C**), *n* = 3). (**D**) CAR-NK cells expressing tEGFR and the mock control were incubated with or without 20% human serum from two different donors and 10 μg/mL cetuximab for 24 h at 37 °C. Cells were analyzed by cell titer glow assay. Mean values ± SEM are shown. Statistical analyses were performed using a two-way ANOVA with Tukey’s multiple comparisons test (**A**,**B**) and ordinary one-way ANOVA with the Kruskal–Wallis comparison test (**C**,**D**). * *p* < 0.05, ** *p* < 0.01, *** *p* < 0.001, **** *p* < 0.0001; ns, non-significant.

To investigate the function of CAR-expressing NK cells, they were first enriched using a Tyto cell sorter (Miltenyi Biotec, Bergisch Gladbach, Germany). To do this, NK cells were labeled with Glypican-3-FITC, CD56-APC, and CD45-PE and sorted, obtaining >95% purity (Figure S4). Mock controls were sorted only based on CD56 expression. Sorted CAR-NK cells were tested for the release of cytokines, addressing, in particular, IL-15 and IFN-α, the two soluble factors subcloned in the vector. Additionally, IL-2 and IFN-γ were also quantified, being involved in the NK-mediated anti-tumor response [43,44,45]. As shown in Figure 3A, both CAR-NK cells released enhanced levels of IL-15 and IL-2 compared to control and mock NK cells. Interestingly, only CAR-GPC3-IL15-IFNα NK cells produced significantly higher amounts of the cytokine IFN-α, as one would expect. No differences were observed in IFN-γ production. To determine whether CAR-NK cells specifically recognized and killed GPC3+ HCC cell lines, we challenged them with common HCC cell lines, such as Huh7, HepG2, and Hep3B. First, we ascertained that these HCC cells expressed GPC3 (Figure S5A). Compared to the mock control, both CAR-GPC3-IL15 and CAR-GPC3-IL15-IFNα showed significantly enhanced killing activity against the GPC3+ Huh7, HepG2, and Hep3B cell lines (Figure 3B). To better ascertain the role of the engineered CAR and to show that killing was restricted by GPC3 engagement, we avoided using human cell lines that were simply negative for the GPC3 antigen and opted for the xenogeneic Chinese hamster ovary cell line engineered to stably overexpress human Glypican-3 (Human GPC3 Stable Cell Line—hGPC3/CHO-K1). As a control, wild-type CHO-K1 negative for GPC3 were used. Specific and stable expression of GPC3 was confirmed by flow cytometry (Figure S5B). As expected, we observed that wild-type CHO-K1 cell lines were negligible to NK cells, being xenogeneic cells (Figure 1C; control and mock). By contrast, ectopic expression of GPC3 conferred susceptibility to CAR-NK cells, as evidenced by the increased cytolysis of hGPC3/CHO-K1 induced by both CAR-GPC3-IL15 and CAR-GPC3-IL15-IFNα NK cells, thus confirming the specificity of the killing driven by cognate antigen (Figure 3C). Importantly, CAR-GPC3-IL15-IFNα NK cells showed stronger killing function than CAR-GPC3-IL15 NKs as demonstrated by the **** *p* at any E:T ratios tested. By contrast, CAR-GPC3-IL15 NK cells scored **** *p* < 0.0001 only at the highest E:T ratios, 10:1 and 20:1. We could observe that both constructs mediated potent and specific cytotoxicity against GPC3-CHO targets, as they both expressed the CAR construct, the engagement of which triggers CD28, 4-1BB, and CD3ζ signaling, providing strong activation. Mock control and parental NK cells did not respond to either hGPC3/CHO-K1 or wild-type CHO-K1 targets, further strengthening the GPC3-specific recognition of the CAR. Our data indicate that the anti-tumor activity of CAR-GPC3-engineered NK cells was dependent on antigen expression on the cell surface.

### 2.4. Cetuximab-Mediated Depletion of CAR-GPC3-NK Cells In Vitro

To confirm that the tEGFR safety switch was both functional and properly exposed on the cell surface, we assessed the expression of the tEGFR incorporated into the fourth-generation anti-GPC3 CAR constructs. This element enables selective cell elimination through anti-EGFR antibody-mediated targeting. Surface tEGFR expression was evaluated on Tyto-sorted CAR-GPC3-IL15 and CAR-GPC3-IL15-IFNα NK cells (Figure S4). Next, we evaluated whether these CAR-NK cells could be lysed upon treatment with the EGFR-specific monoclonal antibody cetuximab. Using an in vitro complement-dependent cytotoxicity assay, we observed that cetuximab alone did not induce cell lysis without serum [29]. However, in the presence of cetuximab and human serum, tEGFR-expressing CAR-NK cells were effectively depleted (Figure 3D). As expected, mock control NK cells, lacking tEGFR expression, were resistant to cetuximab-mediated lysis, confirming the specificity of this depletion mechanism.

## 3. Discussion

Hepatocellular carcinoma, the most common primary liver cancer, carries a poor prognosis due to rising incidence and mortality rates. Surgery remains the first-line treatment for HCC, but the 5-year recurrence rates for patients with HCC after surgery still average between 50% and 70%. Current treatments, particularly for early-stage HCC patients who are ineligible for standard care and those with advanced disease, remain inadequate. This underscores the urgent need for innovative therapeutic strategies [46]. Glypican-3 is overexpressed in approximately 80% of HCC cases and is absent in healthy liver tissue, making it an ideal target for CAR-based immunotherapies. Previous studies have indicated that GPC3 expression is predominantly restricted to certain malignancies such as HCC, ovarian cancer, and yolk sac tumors, with limited or no expression in cancers originating from the gut or lung. This makes GPC3 an ideal and highly selective target for the CAR-NK cell therapy described in this study [2]. With over 20 candidates in clinical trial, GPC3 is a promising target for the treatment of HCC, leveraging cellular therapeutics such as GPC3-redirected CAR-T [16,47], CAR-NK [48], and CAR-iNKT [49] cells but also noncellular reagents like bispecific antibodies engaging T [50,51,52] and NK cells [53] as well as radiopharmaceuticals [54] and GPC3-derived peptide vaccines [46,55].

Glypican-3-CAR T cells have previously been developed as second-generation constructs [14,56], subsequently corroborated by the co-secretion of soluble cytokines like IL-15 and/or IL-21 for the improvement of anti-tumor function [57]. More recently, GPC3-CAR-T cells were engineered to co-express IL-21 and CXCL9, which, alongside blockade of the checkpoint inhibitor PD-1, were shown to offer a combo of increased cytokine secretion, proliferation, chemotaxis, and overall ameliorated anti-tumor activity [58]. Recently, GPC3-CAR-T cells have also been tested in phase I clinical trials, showing promising results [16,47,59,60,61]. CAR-iNKT cells represent an innovative alternative to CAR-T cells by presenting advantages for allogeneic applications in cellular therapy. A recent preclinical study demonstrated that GPC3-specific iNKT cells suppressed tumor progression, promoted survival, and lacked toxicity for tumor-free organs [49]. Compared to T cells, CAR-NK cells offer several distinct advantages. First, NK cells possess an inherent tumor-killing function that is independent of antigen presentation, allowing for broad-spectrum targeting of cancer cells. Their activation is not restricted by human leukocyte antigen (HLA) matching, thus simplifying manufacturing as an “off-the-shelf” therapy. NK cells do not trigger graft-versus-host disease and exhibit a reduced risk of CRS, making them safer compared to CAR-T cell therapies. Additionally, NK cells have a transient lifespan, which minimizes concerns related to prolonged on-target, off-tumor toxicity [62].

Engineering CAR-NK cells from primary NK cells represents the gold standard for immunotherapy. However, the procurement of sufficient peripheral NK cells is technically challenging in many laboratories, involving the isolation and expansion of NK cells from peripheral blood, umbilical cord blood, induced pluripotent stem cells (iPSC), or NK-92 cell lines [63]. Lately, NK cells derived from iPSCs have emerged in the field of cell therapeutics as they can be cultured on a large scale and manufactured off the shelf. In addition, these cells can be transduced more easily than blood-derived primary NK cells, presenting advantages for CAR manufacturing [64]. Kaneko’s group used HLA-homozygous iPSCs to derive NK cells expressing a GPC3-CAR, demonstrating efficacy for the treatment of GPC3+ ovarian cancer [65]. NK-92 cell lines have been explored as CAR-NK platforms [13,66] and have offered insights, for example, into the most strategic routes of cell administration for the achievement of the best therapeutic outcome [66]. However, they suffer from limitations such as poor in vivo persistence and reliance on irradiation, which hinder their clinical application [67,68]. For instance, clinical trials using irradiated NK-92 cells in refractory or relapsed AML patients demonstrated only transient effects [69,70]. GPC3-targeted CAR-NK cells have shown robust anti-tumor activity both in vitro and in vivo, displaying efficient tumor infiltration, suppression of cancer cell proliferation, and induction of apoptosis. Strategies aimed at counteracting the immunosuppressive liver microenvironment have further strengthened the therapeutic potential of GPC3-directed CAR-NK approaches [13].

In this study, we demonstrate that CAR-GPC3-NK cells exhibit potent, specific anti-tumor activity, suggesting their potential as a synergistic clinical option, alongside targeting checkpoint inhibitors [71]. Our laboratory has optimized a protocol to isolate and expand primary human NK cells starting from the liver perfusate, achieving clinically relevant numbers of cells with robust transduction rates for CAR constructs for large-scale CAR-NK cell manufacturing [72]. Owing to the innate characteristics of NK cells, obtaining high levels of transduction is cumbersome [73]. Through the optimized protocol described in this study, we achieved transduction rates of 22.60–47% for CAR-GPC3-IL15 constructs (mean: 36.03% ± 9.8) and 16–41% for CAR-GPC3-IL15-IFNα (mean: 30.3% ± 9.7) by day 5 post-transduction. While the percentage of CAR-expressing NK cells decreased by day 10, the mean fluorescence intensity (MFI) remained high (~40,000), indicating sustained CAR construct expression. This decrease is not due to a loss of CAR expression at the single-cell level, but rather to a shift in the composition of the NK-cell population. As a matter of fact, primary NK cells are heterogeneous and contain subsets with different proliferative capacities. Moreover, following transduction, CAR-positive NK cells tend to proliferate more slowly than non-transduced cells, likely because lentiviral engineering and cytokine co-expression impose an additional metabolic burden. As a result, non-transduced NK cells could expand more rapidly during culture, reducing the overall percentage of CAR-positive cells without affecting CAR density on the cells that remained positive. Our engineered CAR-GPC3-NK cells demonstrated significantly enhanced cytolytic activity against GPC3+ HCC cell lines compared to mock controls. Specificity was confirmed using a xenogeneic CHO-K1 cell line derived from Chinese hamster ovary cells. While primary NK cells showed minimal response against GPC3-negative CHO-K1 cells, CAR-NK cells exhibited significantly enhanced cytotoxicity when challenged with hGPC3/CHO-K1 cells expressing high GPC3 levels.

The fourth-generation CAR constructs used in this study were designed to secrete transgenic soluble IL-15 upon CAR engagement, promoting NK cell homeostasis and function. One construct was additionally engineered to secrete IFN-α, a cytokine previously demonstrated to potently enhance NK cell anti-tumor and anti-viral activity in vitro and in vivo [33,34]. CAR-GPC3-IL15 and CAR-GPC3-IL15-IFNα NK cells released significantly higher IL-15 levels when exposed to GPC3+ HCC targets compared to controls. Notably, IFN-α secretion was exclusively observed in CAR-GPC3-IL15-IFNα cells, confirming functional expression of the engineered constructs. The possibility to control the release of IFN-α results in spatially confined and transient on-target cytokine exposure, thus limiting the side effects known to be associated with the systemic administration of IFN-α. The redirection of NK effector cells is an area of growing interest due to their ability to target multiple tumor antigens, to enhance in vivo proliferation and survival, and to increase infiltration of immune-deserted “cold tumors.” For example, IL-15 and IFN-α secretion may overcome resistance mechanisms in solid tumor microenvironments, as demonstrated by others [43,47]. Chen et al. described GPC3-CAR-NK cells co-expressing soluble PD-L1, highlighting further advancements in this field [43,48]. Recently, CD147 has emerged alongside GPC3 as a promising therapeutic target in liver cancer. Both CD147-CAR-T and CD147-CAR-NK cells have been shown to eliminate HCC cell lines and xenograft tumors in vitro and in vivo [74]. More recently, CD147-CAR-NK cells were reported to offer superior safety compared to CAR-T cells, maintaining strong on-target/off-tumor activity while causing minimal toxicity to CD147^+^ healthy tissues. These findings further underscore the potential safety advantages of CAR-NK therapies in clinical applications, which require robust safety mechanisms to mitigate severe side effects [75]. To this end, both CAR constructs in this study incorporated the tEGFR suicide gene, enabling ex vivo screening, flow cytometry, and in vivo tracking. In our study, cetuximab, a clinically approved anti-EGFR antibody, effectively mediated complement-dependent depletion of CAR-NK cells without affecting mock NK cells [29,30].

This study underscores significant advancements in CAR-NK technology, supporting its potential as a therapeutic option for HCC. The findings suggest that CAR-GPC3-NK cells could serve as an effective, targeted cancer treatment, addressing many limitations associated with CAR-T cells. Future efforts should focus on enhancing CAR-NK cell persistence and function, exploring combination therapies, and scaling production for clinical applications.

## 4. Materials and Methods

### 4.1. Cells and Culture Conditions

Leucocytes were isolated from the perfusion fluid of liver explants, as previously described [72,76,77]. Primary CD3-CD56+ NK cells were isolated from liver perfusate buffy coats using the human NK cell isolation kit (Miltenyi Biotec, Bergisch Gladbach, Germany, Europe). Cell isolates with <90% viability, as assessed by trypan blue exclusion test, and <95% purity were excluded from the study. NK cells were cultured immediately after isolation in MACS NK medium (Miltenyi Biotec, Bergisch Gladbach, Germany) supplemented with 500 IU/mL IL-2 (Proleukin, Chiron, Emeryville, CA, USA) and 20 ng/mL IL-15 (Miltenyi Biotec, Bergisch Gladbach, Germany) in the presence of NK activation/expansion beads for five days (Miltenyi Biotec, Bergisch Gladbach, Germany), or they were cryopreserved. The concentrations of IL-2 and IL-15 were chosen based on titration assays (IL-2: 250–1000 IU/mL; IL-15: 10–30 ng/mL) as previously described [33,34]. Where indicated, NK cells were further stimulated overnight with IL-2/IL-15 or 1 µg/mL IFN-α (Origene, Herford, Germany, Europe) or 10ng/mL IL-12 (Miltenyi Biotec, Bergisch Gladbach, Germany) and 100ng/mL IL-18 (D.B.A., Segrate, Italy). Before downstream applications, NK cells were thoroughly washed. The Huh7 cells were a kind gift of Prof. R. Bartenschlager with the authorization of Apath L.L.C., NY and were kept in culture with High Glucose Dulbecco Minimum Essential Medium (DMEM) (Lonza, Milan, Italy) HepG2 and Hep3B2.1-7 cells were purchased from ATCC (LGC Standards, S.r.L., Milan Italy) and kept in culture with Eagle’s Minimum Essential Medium (EMEM) (Lonza, Milan, Italy). The GPC3 Stable CHO Cell Line was purchased from Abeomics (CliniSciences, Guidonia Montecelio, Italy). The CHO-K1 cell line was purchased from Merck (Merck Life Sciences S.r.l., Milan, Italy) and maintained in culture with Ham’s F12 media (Merck Life Sciences S.r.l., Milan, Italy).

### 4.2. Flow Cytometry

Aliquots of 0.5 × 10^6^ NK cells were analyzed for surface markers using a panel of prediluted fluorochrome-conjugated anti-human monoclonal antibodies: FITC-CD3, Streptavidin-APC-Cy7 (BD Biosciences, Milan, Italy). PE-CD45 and APC-CD56 (eBioscience, San Diego, CA, USA). FITC-Glypican-3 (D.B.A., Segrate, Italy). APC-Anti-Glypican-3 (Sino Biological, ProdottiGianni, Milan, Italy). Biotin-EGFR (Cetuximab Biosimilar) (R&D Systems, Bio-Techne, Milan, Italy). Samples were run on a 16-color FACS Celesta SORP flow cytometer (Becton Dickinson, San Jose, CA, USA) and data were analyzed with FlowJo software (version 10).

### 4.3. Plasmid and Viral Production

We designed two 4th generation CAR constructs specific to Glypican-3 tumor antigen to be expressed by NK cells. The vectors were designed as follows: (1) Lenti-anti-GPC3-h(28BBζ)-3rd-CAR-EGFRt-IL15 (EF-1alpha promoter-Signal peptide-scFv-CD8 hinge-CD28 TM intracellular-4-1BB-CD3zeta-T2AEGFRt-KpnI-IL15-XbaI) (herein indicated as CAR-GPC3-IL15); (2) Lenti-anti-GPC3-h(28BBζ)-3rd-CAR-IFNα-EGFRt-IL15 (EF-1alpha promoter-Signal peptide-scFv-CD8 hinge-CD28 TM intracellular-4-1BB-CD3zeta-T2AIFNα-T2A-EGFRt-KpnI-IL15-XbaI) (herein indicated as CAR-GPC3-IL15-IFNα). Both plasmids were commissioned to Creative Biolabs (Shirley, NY, USA). Lentiviral vector particles were produced by transfection of HEK 293T (CRL-3216™, ATCC, LGC Standards S.r.l., Sesto San Giovanni, Italy). GFP plasmid control TWEEN was used to control the efficiency of transfection. LentiArt™ Virus Packaging Kit (Creative Biolabs, Shirley, NY, USA) was used as a packaging tool with Lipofectamine3000 transfection reagent (Life Technologies, Monza, Italy). Plasmids were purified using EndoFree Plasmid Maxi Kit (QIAGEN, M-MICROMED S.r.l.s., Catania, Italy), as detailed elsewhere [78,79]. Plasmids pCAR-GPC3-IL15 and pCAR-GPC3-IL15-IFNα diagnosed by digestion with restriction enzymes NotI and XhoI (NEB, Euroclone S.p.A., Pero, MI, Italy) and ran on 1% Agarose gel in TAE buffer (Sigma-Aldrich, Merck, Milan, Italy). Markers were Purple 1Kb ladder and Purple 100 bp ladder (NEB, Euroclone S.p.A., Pero, MI, Italy). Bands expected: 2100 and 8100 bp for pCAR-GPC3-IL15-IFNα and 2100 and 7500 bp for pCAR-GPC3-IL15. Briefly, 24 h before transfection, 4 × 10^6^ HEK 293T cells were seeded on a 10 cm culture dish in DMEM supplemented with 10% FBS and 1% L-glutamine. For transfection, cells were washed twice with 5 mL of PBS, and medium was exchanged with 10 mL of Optimem (Life Technologies, Monza, Italy) containing Lipofectamine3000 (Thermo Fisher Scientific, Rodano, Italy), LentiArt™ Virus Packaging Kit (Creative Biolabs, Shirley, NY, USA) and one of the plasmids containing the construct (TWEEN, CAR-GPC3-IL15, or CAR-GPC3-IL15-IFNα) following manufacturer’s instructions. The medium was changed after 6–12 h. Supernatants containing the viral particles were collected 72 h after transfection and filtered through a 0.45 μm filter, concentrated using Lentivirus concentration solution (Origene, TEMA Ricerca, Castenaso, Italy), aliquoted, and stored at −80 °C until use.

### 4.4. Viral Transduction

NK cell transduction was performed using the viral supernatant and Vectofusin (10 µg/mL, Miltenyi Biotec, Bergisch Gladbach, Germany) on untreated 24 multi-well plates (CLEARLine^®^, Biosigma, Cona, VE, Italy) previously coated with 7.5 µg/mL RetroNectin^®^ Recombinant Human Fibronectin Fragment (Takara, Diatech Lab Line Srl, Jesi, AN, Italy). After adding the viral supernatant, 1 × 10^5^ primary NK cells were added in each well, followed by spinoculation at 1800 rpm for 1 h at 32 °C. NK cells were cultured with a lentiviral vector for 24 h. We previously tested four different ratios of viral supernatant to culture volume (1:1, 1:2, 1:5, and 1:10). Among these conditions, the 1:5 dilution consistently yielded the best balance between transduction efficiency, cell viability, and CAR expression stability. The cell culture medium was replaced with a fresh, complete cell culture medium. Transduction efficiency was determined by flow cytometry on days 5 and 10 post-transduction.

### 4.5. Cell Sorting

Transduced CAR-NK cells were then stained with anti-CD45-PE, anti-CD56 APC, and Glypican-3-FITC and were sorted on the Tyto (Miltenyi Biotec, Bergisch Gladbach, Germany) using CD45-PE as the cell trigger and CD56-APC/Glypican-3-FITC as the cell speed channel. All samples were >98% pure as determined by flow cytometry analysis.

### 4.6. Cytotoxicity Assay

A four-hour chromium-51 (51Cr) release assay (Perkin Elmer Italia, Milan, Italy) was used to measure the specific lysis of target cells exposed to decreasing ratios of NK cells (E:T ratios: 20:1, 10:1, 5:1, 2,5:1, or 1:1), as previously described [33,34]. The percent of target cell lysis was calculated as follows: (mean experimental counts per minute (cpm) − mean spontaneous cpm)/(mean maximum cpm − mean spontaneous cpm) × 100%

### 4.7. Multiplex Analysis of Cytokines

Human cytokines IL-2, IL-15, IFN-α, and IFN-γ in CAR-NK cell conditioned media were assessed by ProcartaPlex MixMatch Magnetic bead kit (Affymetrix, Prodotti Gianni, Milan, Italy) following the manufacturer’s instructions. Results were obtained with Luminex^TM^ 200 Systems (Luminex Corporation, Austin, TX, USA). Fluorescence intensity (FI) data from the assays were used for further analysis.

### 4.8. Analysis of AFP Levels

AFP levels in the supernatant of cultured cells were measured by on the Siemens Dimension^TM^ Vista 1500 Analyzer (Siemens Healthcare Diagnostics S.r.l., Milan, Italy) following the manufacturer’s instructions. Samples were centrifuged to remove the remaining cells and possible cell debris before testing the AFP concentration.

### 4.9. Cetuximab-Mediated Depletion of CAR-NK Cells

Complement-dependent cytotoxicity was performed by incubating CAR-NK cells (2 × 10^6^/mL) with or without 20% human serum from two different donors in the presence of 10 μg/mL cetuximab. After 24 h at 37 °C, viable cells were detected by CellTiter-Glo^®^ Luminescent Cell Viability Assay (Promega Italy, Milan, Italy) following the manufacturer’s instructions. Plates were read on the Spark microwell plate reader (Tecan, Mannedorf, Switzerland).

### 4.10. Statistical Analyses

The statistical analyses considered both technical and biological replicates. Specific tests are described in the legends and were performed using GraphPad Prism software, v9-10. Statistical tests were considered significant when the *p*-value was less than 0.05 (* *p* < 0.05; ** *p* < 0.01; *** *p* < 0.001; **** *p* < 0.0001; not significant, ns).

## 5. Patents

Nk-mediated immunotherapy and uses thereof, WO2018099988A1.

## Data Availability

The original contributions presented in the study are included in the article/Supplementary Materials; further inquiries can be directed to the corresponding author/s.

## Supplementary Materials

The following supporting information can be downloaded at https://www.mdpi.com/article/10.3390/ijms262411892/s1.
