# Supplementary material for: Glypican-3-Specific CAR NK Cells Co-Secreting IL-15 and IFN-α Have Increased Anti-Tumor Function Versus Hepatocellular Carcinoma In Vitro
Source: Int J Mol Sci. 2025 Dec 10;26(24):11892. doi: 10.3390/ijms262411892 (PMC12733051; doi:10.3390/ijms262411892)
Supplement: Supplementary file 1 [file ijms-26-11892-s001.zip › ijms-4014092-supplementary.pdf]

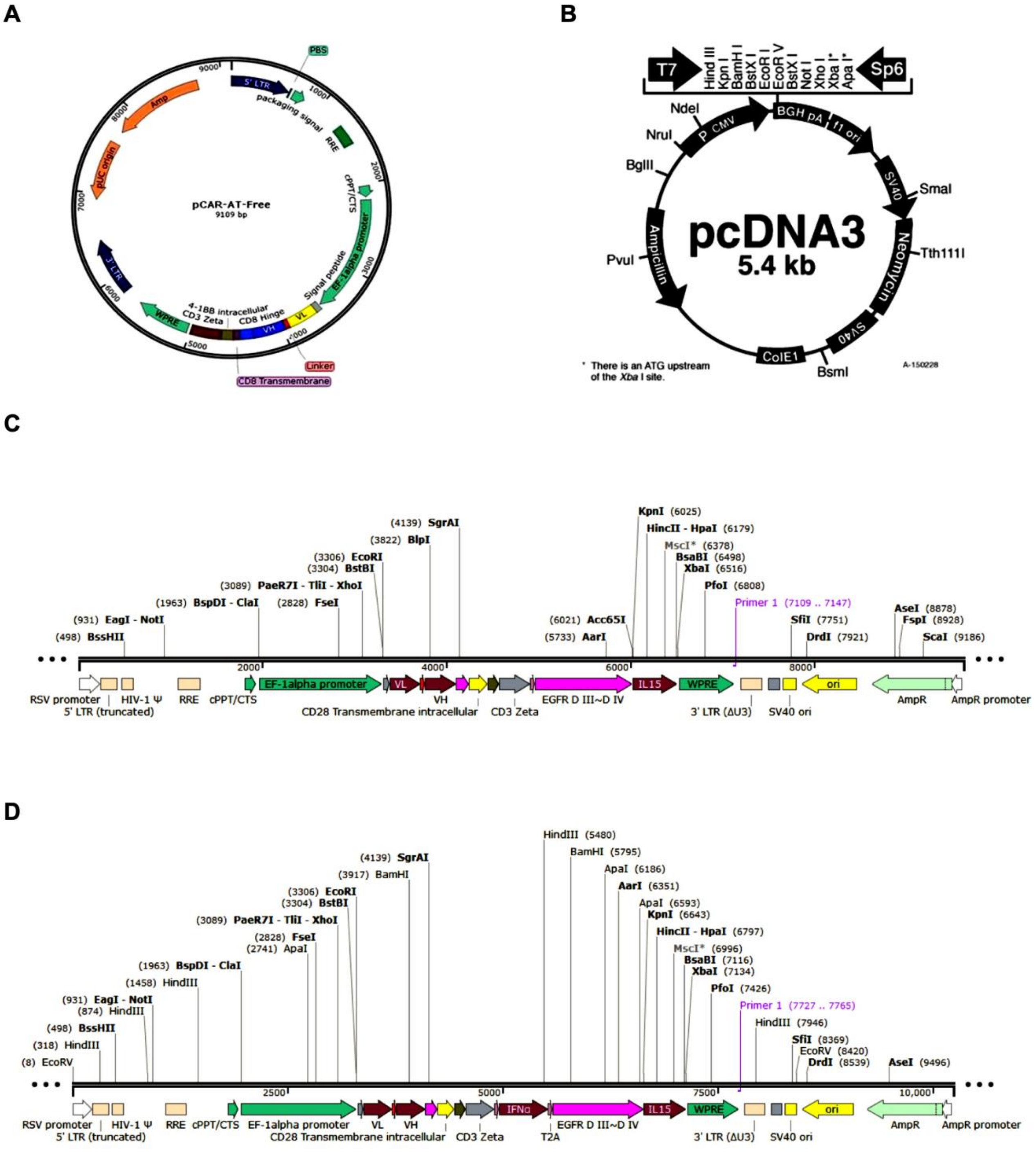

Supplementary Figure S1: Schematic representation of GPC3-specific CAR construct and the lentiviral vector. **(A, B)** GPC3-CAR expression plasmid for lentiviral preparation. **(C, D)** The construct sequence consisted of a signal peptide, hinge region, transmembrane domain, and intracellular region as follows: Lenti-anti-GPC3-h(28BBζ)-3rd-CAR-EGFRt-IL15 (EF-1alpha promoter-Signal peptide-scFv-CD8 hinge-CD28 transmembrane intracellular-4-1BB-CD3zeta-T2AEGFRt-KpnI-IL15-XbaI) **(C)** and Lenti-anti-GPC3-h(28BBζ)-3rd-CAR-IFNα-EGFRt-IL15 (EF-1alpha promoter-Signal peptide-scFv-CD8 hinge-CD28 transmembrane intracellular-4-1BB-CD3zeta-T2AIFNα-T2A-EGFRt-KpnI-IL15-XbaI) **(D)**.

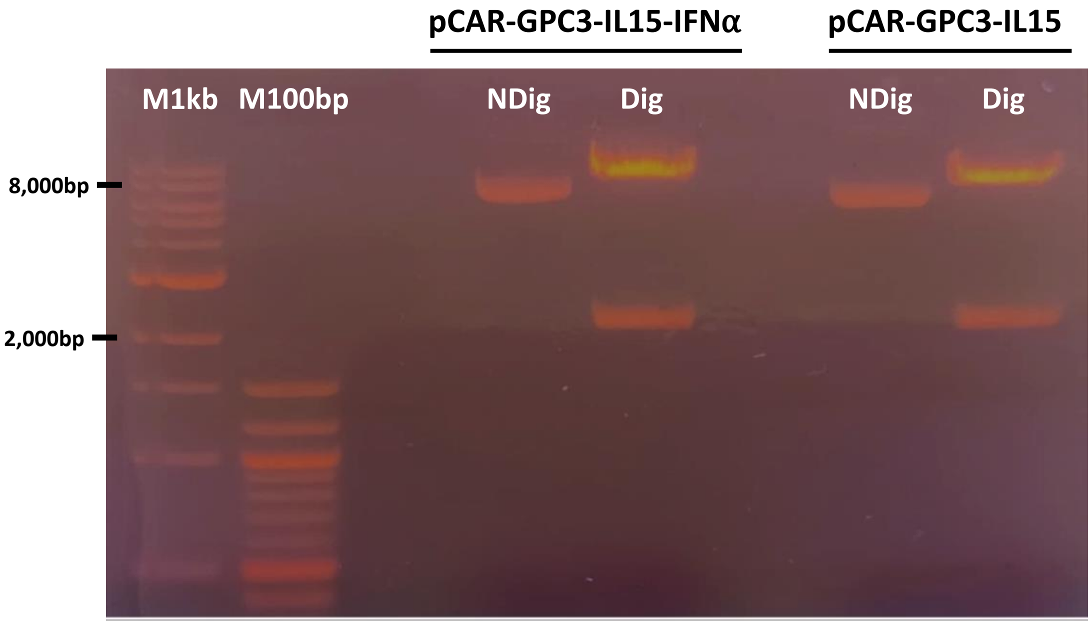

Supplementary Figure S2: Diagnostic digestion of plasmids pCAR-GPC3-IL15-IFNα and pCAR-GPC3-IL15 with NotI and XhoI restriction enzymes confirmed the expected size bands for both plasmids, i.e., 2,100 and 8,100 bp for pCAR-GPC3-IL15-IFNα and 2100 and 7500 bp for pCAR-GPC3-IL15. M1kb and M100bp refer to Purple 1Kb ladder NEB and Purple 100bp ladder (NEB), Ndig and Dig refer to non-digested and digested, respectively.

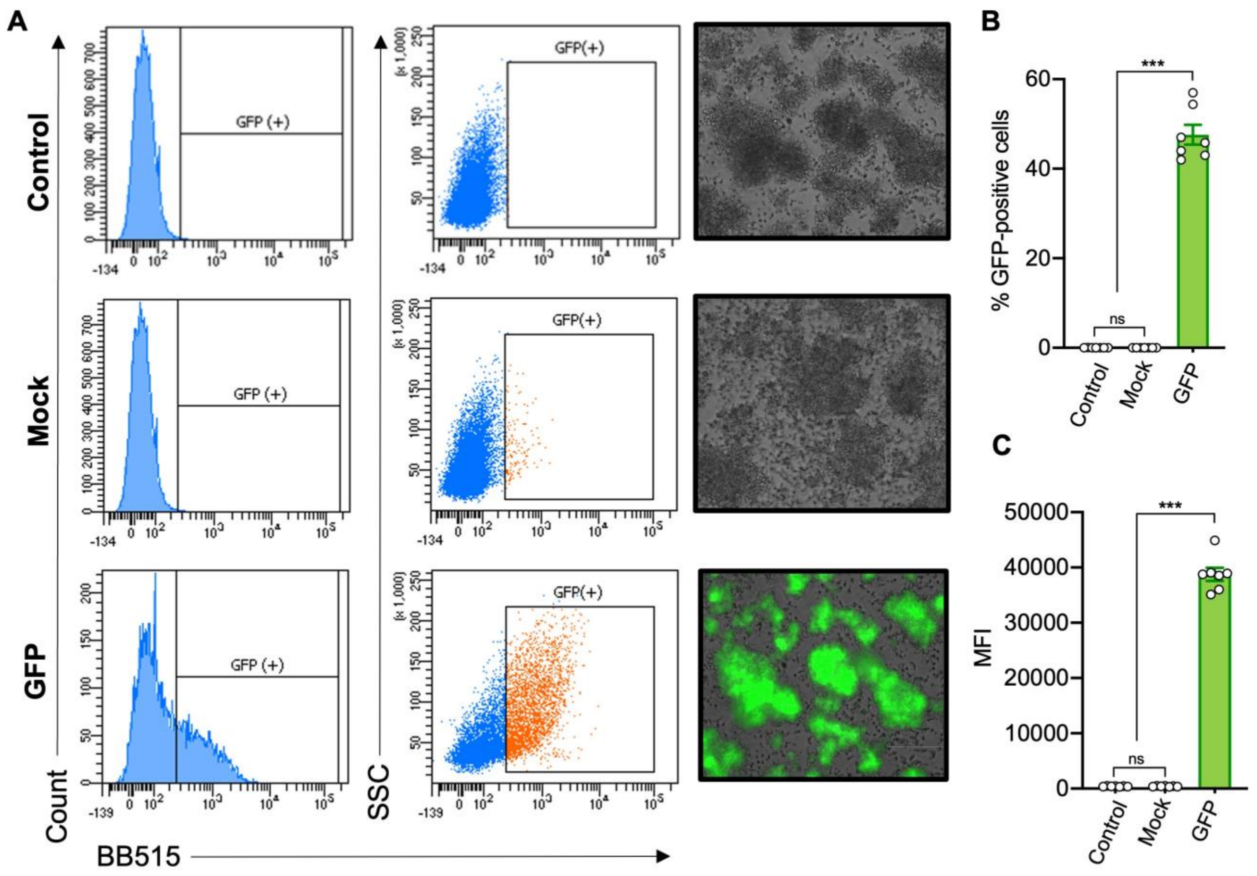

Supplementary Figure S3: (A) Flow cytometric analysis of GFP expression of pure primary NK not transduced (control), transduced with lentiviral vector backbone (mock), or transduced with a plasmid encoding a GFP reporter gene (GFP). NK cells were gated on live cells according to morphology FSC/SSC. Data shown are representatives of seven experiments with similar results. **(B, C).** Efficiency of transduction was traced at day five. Symbols (n = 7) represent the percentage of GFP-positive cells **(B)** and the Mean Fluorescence Intensity (MFI, **C**). Statistical analyses were performed using a two-way ANOVA with the Kruskal-Wallis comparisons test. * $p < 0.05$, ** $p < 0.01$, **** $p < 0.0001$; ns, non-significant.

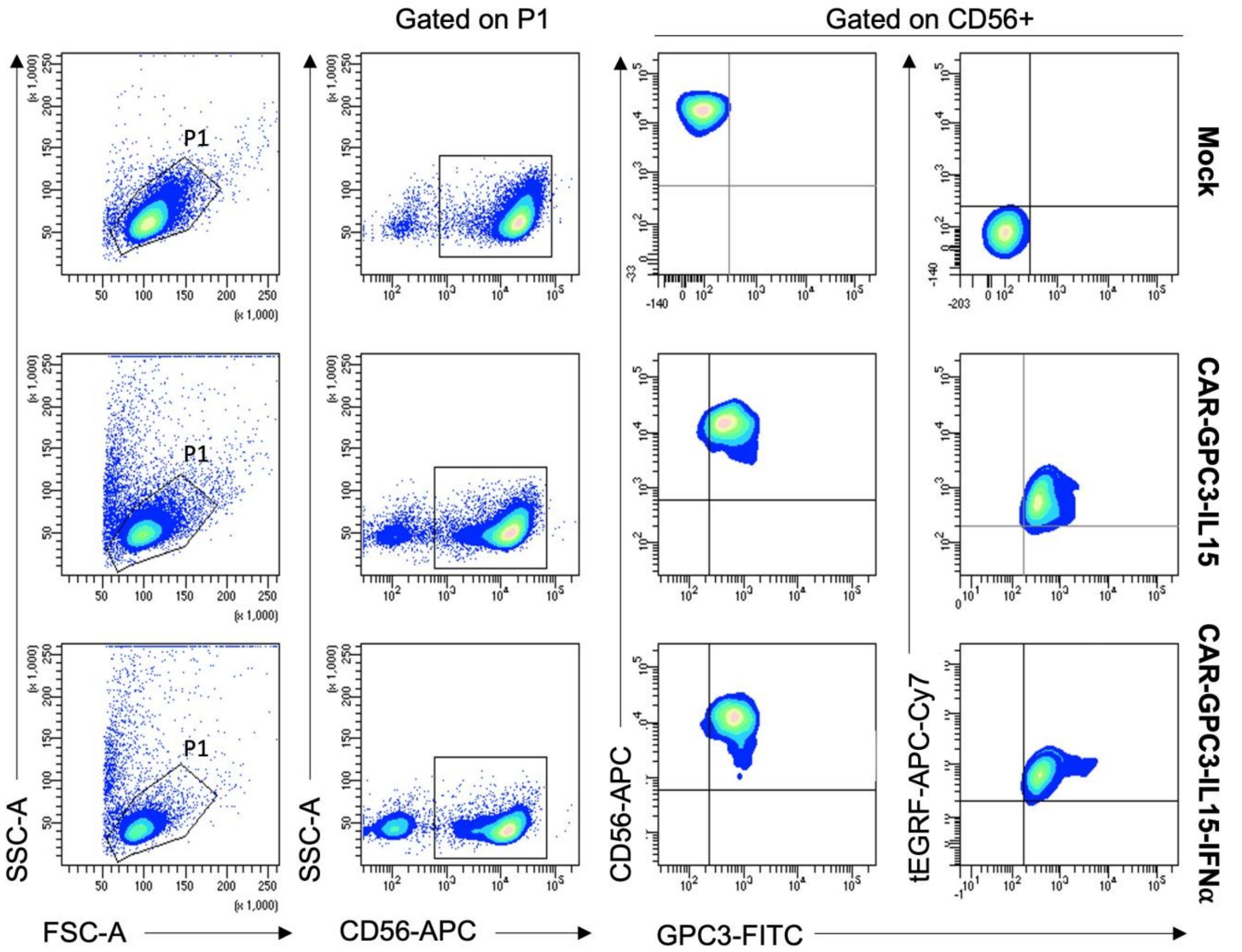

Supplementary Figure S4: Density plots of Tyto-sorted CAR-GPC3-IL15 and CAR-GPC3-IL15-IFNα NK cells stained with CD56-APC, GPC3-FITC and tEGFR-biotin/streptavidin-APC-Cy7. The gating strategy used consisted of gating cells based on the FSC/SSC morphology plot (P1). A second gate was made on CD56+ cells. Density plot shows that sorted CD56+ CAR-NK cells were pure and expressed both GPC3-CAR and tEGFR.

A

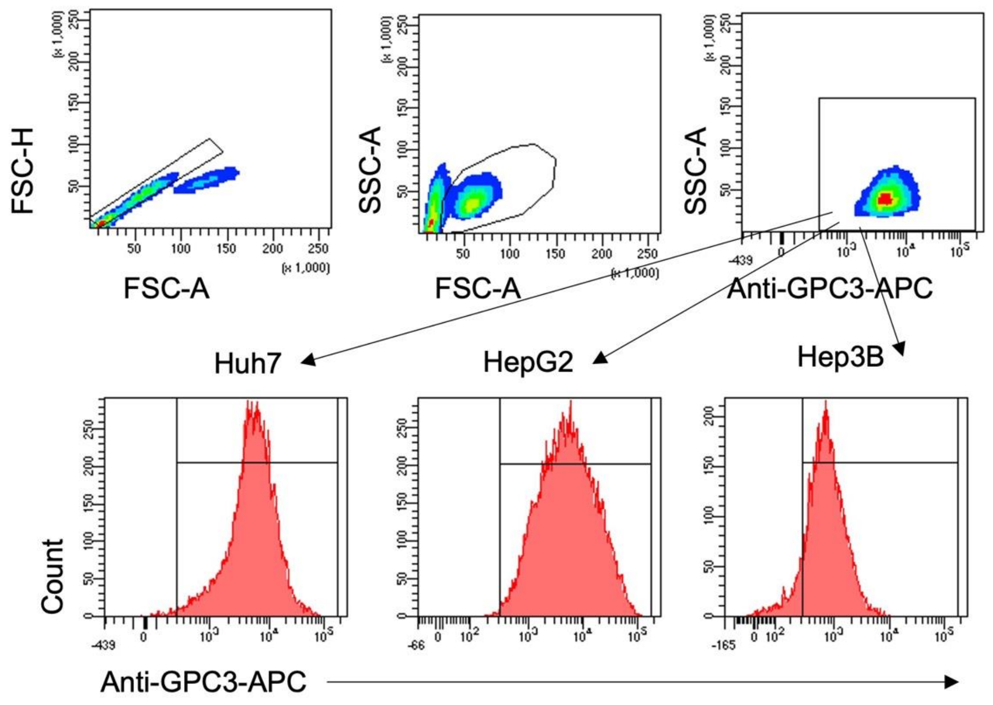

B

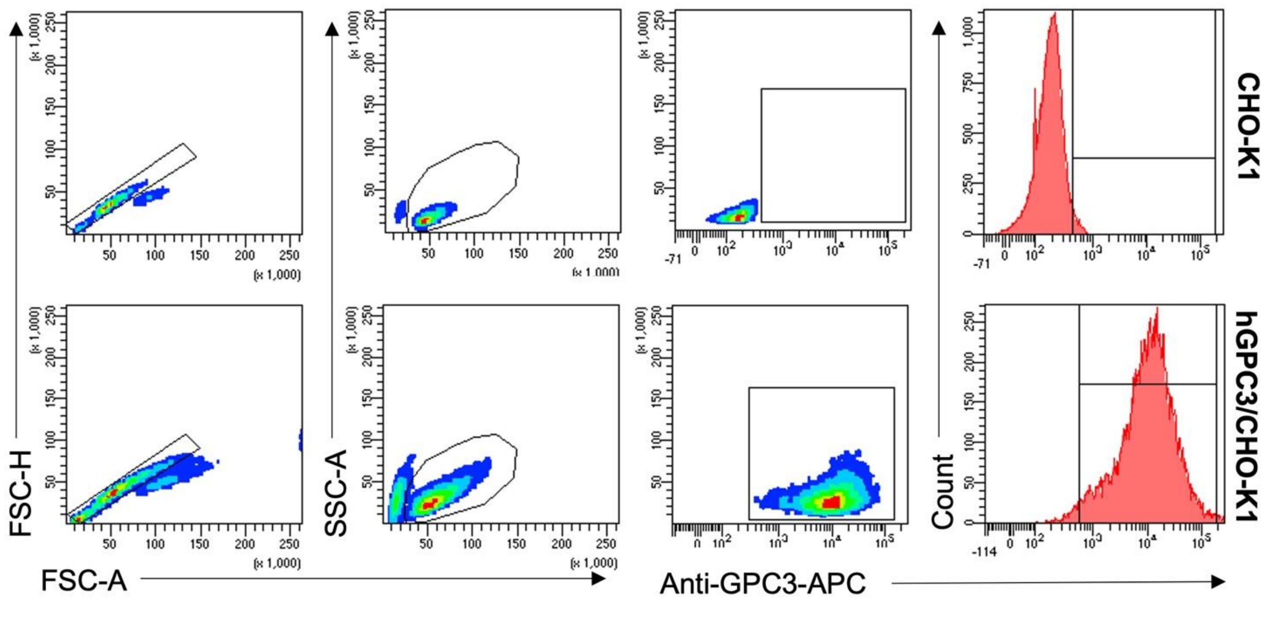

Supplementary Figure S5: GPC3 expression in HCC cell lines. Flow cytometry analysis to detect GPC3 cell surface expression in Huh7, HepG2, and Hep3B cells **(A)** and CHO-K1 and hGPC3/CHO-K1 cells **(B)**.
